# From a Biodegradable Scaffold to a Living Artery: Native Arterial Wall Regeneration Following Hybrid Tissue-Engineered Vascular Grafting

**DOI:** 10.7759/cureus.111186

**Published:** 2026-06-20

**Authors:** Kazuyuki Ishibashi, Mamika Motokawa

**Affiliations:** 1 Cardiovascular Surgery, Ship International Hospital, Dhaka, BGD

**Keywords:** artificial vascular graft, biodegradable scaffolds, biomaterial, cardiovascular tissue engineering, cell seeding, vascular surgery

## Abstract

Background

Long-term patency of small-diameter vascular grafts remains limited by thrombosis, intimal hyperplasia, and compliance mismatch with native arteries. Despite decades of advances in tissue engineering, no artificial graft has yet been developed that can permanently replicate the multiple biological functions of native vessels, including their antithrombotic properties. We hypothesized that a hybrid tissue-engineered vascular graft composed of endothelial cells (ECs), smooth muscle cells (SMCs), and fibroblasts (FCs) would promote vascular regeneration and facilitate the formation of a native artery-like wall following scaffold degradation.

Aim

The aim of this study was to evaluate scaffold degradation, vascular wall remodeling, and SMC phenotypic maturation in hybrid tissue-engineered vascular grafts implanted under arterial hemodynamic conditions.

Methods

Hybrid tissue-engineered vascular grafts were constructed by sequentially seeding autologous ECs, SMCs, and FCs onto a biodegradable lactide/ε-caprolactone scaffold reinforced with bioabsorbable mesh fibers. A total of 12 mongrel dogs were used in this study. Hybrid grafts (5 cm in length) were implanted as carotid artery interposition grafts and harvested after two weeks (n = 4) or eight weeks (n = 4), whereas non-seeded grafts containing extracellular matrix alone served as controls (n = 4). Graft patency, endothelialization, vascular wall organization, scaffold degradation, and SMC phenotype were evaluated by histology, scanning electron microscopy (SEM), transmission electron microscopy (TEM), and morphometric analyses.

Results

All hybrid grafts remained patent throughout the study period, whereas all control grafts failed within one week after implantation. Complete endothelial coverage was maintained in all patent grafts. Graft diameter increased from 5.0 mm before implantation to 6.0 ± 0.67 mm at two weeks and remained stable thereafter (6.4 ± 0.12 mm at eight weeks). Neoarterial wall thickness increased significantly from 50.8 ± 10.0 μm before implantation to 329 ± 133 μm at two weeks, followed by regression to 99.9 ± 28.4 μm at eight weeks (P < .05). Histological analysis demonstrated progressive organization of vascular wall cells into a layered architecture resembling that of native arteries. Most polymer components were no longer detectable at two weeks and had completely disappeared by eight weeks, whereas reinforcing mesh fibers remained present within the regenerated tissue. TEM demonstrated a temporal transition of SMCs from a synthetic phenotype at two weeks to a predominantly contractile phenotype at eight weeks.

Conclusions

Hybrid tissue-engineered vascular grafts promoted rapid endothelialization, progressive vascular wall maturation, and complete replacement of the biodegradable scaffold by organized arterial tissue. The transition from synthetic to contractile SMCs, together with the formation of a native artery-like wall despite scaffold degradation, indicates that the graft functioned as a temporary regenerative template rather than a permanent prosthesis. These findings support the feasibility of generating a living arterial conduit capable of long-term remodeling and potentially continued growth following scaffold resorption.

## Introduction

The development of a small-diameter vascular graft with long-term patency remains one of the most challenging issues in cardiovascular surgery. Although synthetic prostheses such as expanded polytetrafluoroethylene and polyethylene terephthalate (Dacron) have achieved acceptable outcomes in large-caliber arterial reconstruction, their performance in vessels smaller than 6 mm remains unsatisfactory because of thrombosis, intimal hyperplasia, and compliance mismatch [1,2]. Furthermore, currently available prosthetic grafts lack the capacity for growth and remodeling, limiting their application in pediatric cardiovascular surgery [3].

Native blood vessels possess a highly organized trilaminar architecture consisting of endothelial cells (ECs), smooth muscle cells (SMCs), and fibroblasts (FCs). These cellular components interact through extracellular matrix-mediated signaling to maintain vascular homeostasis, preserve mechanical integrity, and regulate thromboresistance [4]. In particular, endothelial cells provide dynamic anticoagulant and antiplatelet functions that cannot be permanently replicated by existing synthetic surface modifications [5].

To overcome the limitations of conventional prosthetic grafts, numerous tissue-engineering strategies have been investigated, including biodegradable polymer scaffolds [6], decellularized vascular conduits [7], cell-seeded grafts [8], and three-dimensional bioprinted vascular constructs [9]. Despite encouraging experimental results, the development of a clinically applicable small-diameter vascular graft capable of maintaining long-term patency while undergoing physiological vascular remodeling remains elusive.

We previously reported that hierarchical reconstruction of a hybrid vascular graft using ECs, SMCs, and FCs facilitated vascular tissue formation in vivo [10]. Building on this concept, we developed a hybrid tissue-engineered vascular graft consisting of autologous ECs, SMCs, and FCs seeded onto a biodegradable scaffold reinforced with absorbable mesh fibers.

The primary objective of this study was to determine whether a biodegradable scaffold could be replaced by a native artery-like wall after implantation under arterial hemodynamic conditions. Specifically, we evaluated scaffold degradation, endothelial preservation, vascular wall organization, changes in graft geometry, and smooth muscle cell phenotypic maturation during neovessel formation. An ideal biodegradable vascular graft should function as a temporary regenerative template and ultimately be replaced by living vascular tissue capable of long-term remodeling and growth. Whether such complete biological replacement can occur in the arterial circulation remains incompletely understood.

## Materials and methods

Experimental design

Twelve adult male mongrel dogs weighing 16-25 kg were used in this study. Autologous ECs, SMCs, and FCs were harvested, expanded in culture, and used to construct hybrid tissue-engineered vascular grafts. The grafts were implanted as carotid artery interposition grafts and explanted after two weeks (n = 4) or eight weeks (n = 4). Non-seeded grafts containing extracellular matrix without cellular components served as controls (n = 4).

Animals were randomly assigned to the two-week or eight-week groups. Histological and morphometric analyses were performed by investigators blinded to the experimental groups.

All procedures were approved by the institutional animal care committee and complied with the Principles of Laboratory Animal Care (Akita University, Akita City, Japan) and the Guide for the Care and Use of Laboratory Animals (National Institutes of Health Publication No. 80-23, revised 1985). The study was conducted and reported in accordance with the ARRIVE (Animal Research: Reporting of In Vivo Experiments) 2.0 guidelines for animal research.

Cell isolation and culture

Animals were sedated with intramuscular ketamine hydrochloride (10 mg/kg) and anesthetized with intravenous sodium thiopental (25 mg/kg as required). Following endotracheal intubation, anesthesia was maintained under mechanical ventilation. Autologous ECs were harvested from the external jugular veins by digestion with 0.1% collagenase for 14 minutes at 37°C. The isolated cells were cultured in Medium 199 supplemented with 20% fetal bovine serum, heparin (25 μg/mL), basic FC growth factor (25 ng/mL), penicillin (50 U/mL), streptomycin (50 μg/mL), and fungizone (2.5 μg/mL). SMCs were isolated from the medial layer of the external jugular vein using an explant technique after endothelial harvesting. FCs were obtained from dermal tissue adjacent to the harvested vein using the same explant method. Both SMCs and FCs were cultured in Medium 199 supplemented with 10% fetal bovine serum and antibiotics. All cell types were used between passages 2 and 3.

Cell characterization

EC identity was confirmed by characteristic cobblestone morphology and uptake of fluorescently labeled acetylated low-density lipoprotein (Dil-Ac-LDL). SMCs were identified by positive immunostaining using the monoclonal antibody hybridoma human fetus clone 35 (HHF-35), which recognizes muscle-specific α- and γ-actin isoforms expressed in smooth and skeletal muscle cells. FCs were negative for both Dil-Ac-LDL uptake and α- and γ-actin staining.

Graft fabrication

The vascular graft was fabricated from a biodegradable copolymer consisting of a 50:50 molar ratio of lactide and ε-caprolactone. The scaffold wall thickness was 500 μm, and the pore size ranged from 20 to 100 μm. Mechanical reinforcement was achieved by external wrapping with a bioabsorbable polyglactin 910 mesh.

Construction of hybrid vascular grafts

**Figure 1:**
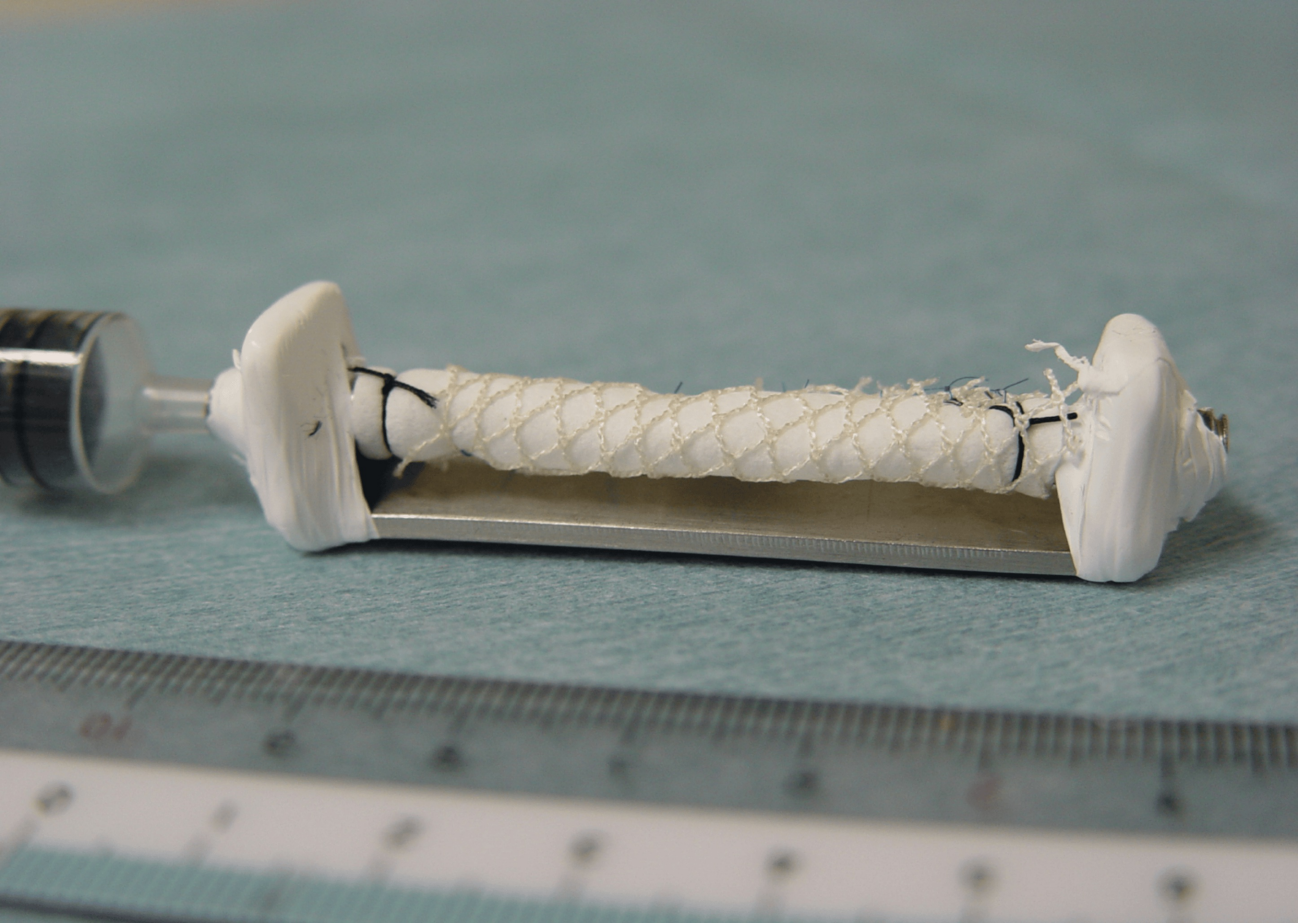
Construction of the hybrid tissue-engineered vascular graft. The biodegradable vascular scaffold was mounted on a custom-designed vascular holder. The outer surface of the scaffold was reinforced with a bioabsorbable mesh to provide mechanical support during vascular remodeling.

Hybrid vascular grafts were constructed according to our previously reported method [10]. An artificial extracellular matrix was prepared by mixing equal volumes of 0.5% type I collagen solution and Medium 199. The scaffold was mounted on a custom-designed vascular holder (Figure 1), impregnated with the collagen matrix, and incubated at 37°C for 10 minutes to allow gel formation. Residual collagen was subsequently removed by rinsing with Hank’s balanced salt solution.

FCs (3.0 × 10⁶ cells) suspended in collagen solution were applied to form the adventitial component of the graft. Subsequently, SMCs (3.0 × 10⁶ cells) suspended in collagen solution were layered onto the FC-containing matrix to create the medial component.

Following maturation of the vascular wall construct, ECs were seeded onto the luminal surface at a density of 6.0 × 10⁵ cells/cm² using a rotational seeding technique. Grafts were maintained in culture for an additional 3 days before implantation.

Construction of control grafts

Control grafts were prepared using the identical biodegradable scaffold and extracellular matrix described above, but without cellular components. The scaffold was impregnated with collagen matrix alone and maintained under the same culture conditions as the hybrid grafts until implantation. The extracellular matrix-only grafts were used as negative controls to determine whether preconstruction of a cellular vascular wall is necessary for maintaining graft-promoting vascular regeneration under arterial hemodynamic conditions. By using the same biodegradable scaffold and extracellular matrix in both groups, we sought to isolate the contribution of vascular wall cells to graft remodeling and long-term structural integrity.

Surgical implantation

After systemic heparinization (200 U/kg), hybrid grafts measuring 5 cm in length were implanted into the carotid arteries of the same animals from which the vascular cells had been harvested. A segment of each graft remaining within the holder was retained for preimplantation histological evaluation. End-to-end anastomoses were performed using continuous 7-0 polypropylene sutures. Neither postoperative anticoagulation nor antiplatelet therapy was administered. Animals implanted with hybrid grafts were sacrificed at predetermined time points of two weeks (n = 4) and eight weeks (n = 4). Control grafts consisting of extracellular matrix-filled scaffolds without cellular components were implanted using an identical protocol. Control animals were monitored daily and euthanized if they reached predefined humane endpoints. All control animals developed rapidly expanding cervical hematomas within one week after implantation and underwent emergency graft explantation.

Tissue harvesting

At explantation, animals received systemic heparinization (200 U/kg). The graft and adjacent native artery segments, including both anastomoses, were excised en bloc. The specimens were gently flushed with physiologic saline solution to remove residual blood and fixed in a solution containing 2% paraformaldehyde, 0.5% glutaraldehyde, and 0.1% tannic acid in 0.1 mol/L sodium cacodylate buffer for eight hours. The specimens were subsequently processed for light microscopy, scanning electron microscopy (SEM), and transmission electron microscopy (TEM).

Scanning Electron Microscopy

Specimens for SEM were post-fixed in 1% osmium tetroxide, dehydrated through graded ethanol solutions, critical-point dried with carbon dioxide, and sputter-coated with platinum. Luminal endothelial coverage was evaluated using SEM images obtained at ×200 magnification.

Transmission Electron Microscopy

Specimens for TEM were post-fixed in 1% osmium tetroxide, stained en bloc with 2% uranyl acetate, dehydrated in graded ethanol, and embedded in Spurr resin. Ultrathin sections were examined to assess vascular wall ultrastructure and smooth muscle cell phenotype.

Morphometric analysis

Graft diameter and neo-arterial wall thickness were measured before implantation and after explantation. Post-implantation measurements were obtained at the proximal, middle, and distal portions of the graft. Measurements were performed using digital image analysis, and the mean of eight measurements was used for statistical analysis. For quantitative assessment of endothelial coverage, specimens were obtained from 10 predetermined sites on each graft. The percentage of luminal surface covered by endothelial cells was calculated from SEM images using planimetric image analysis. For ultrastructural analysis, three representative tissue blocks were selected from each graft. SMCs were classified as synthetic or contractile phenotypes according to established ultrastructural criteria. The presence or absence of residual polymer scaffold and mesh fibers was assessed by light microscopic examination of histological sections at each implantation period.

Statistical analysis

Continuous variables are expressed as mean ± standard deviation. Comparisons among groups were performed using one-way analysis of variance (ANOVA). When a significant overall difference was detected, pairwise comparisons were performed using the two-tailed Student’s t-test. A value of P < .05 was considered statistically significant.

## Results

Construction of hybrid vascular grafts

**Figure 2:**
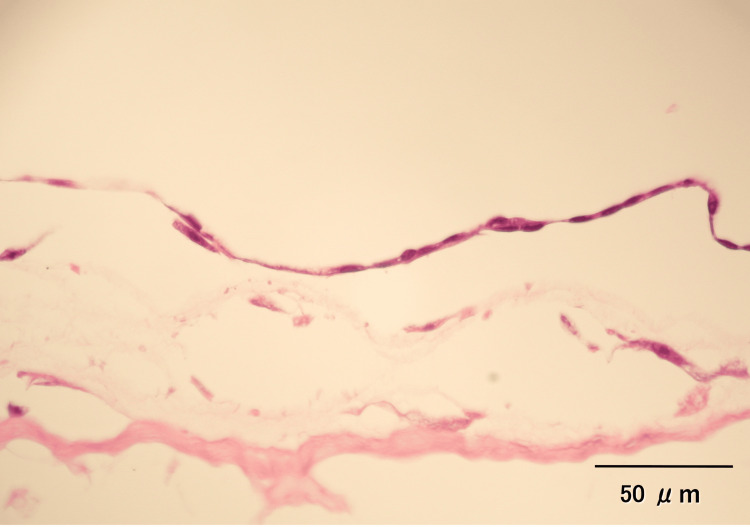
Histological appearance of the hybrid vascular graft before implantation. Light microscopic section of the preimplantation graft; the luminal surface was covered by a confluent endothelial cell monolayer. The graft wall contained abundant smooth muscle cells and fibroblasts embedded within a loose collagen extracellular matrix.

**Figure 3:**
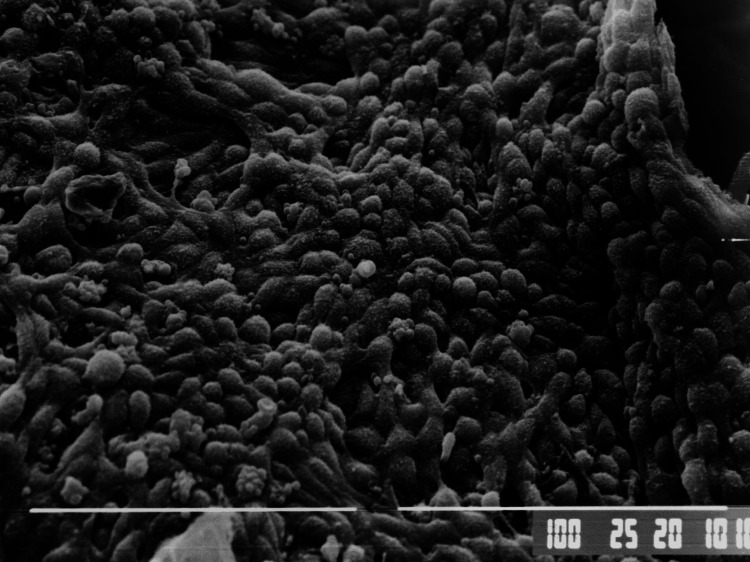
Scanning electron microscopic appearance of the luminal surface before implantation. The luminal surface was almost completely covered by endothelial cells exhibiting a characteristic cobblestone morphology without a preferential orientation.

Histological examination of preimplantation grafts demonstrated successful incorporation of vascular wall cells within the collagen matrix (Figure 2). The density of incorporated SMCs and FCs was 1.8 ± 0.27 × 10² cells/mm². After an additional three-day incubation period, SEM confirmed nearly complete endothelial coverage of the luminal surface in all grafts. The endothelial cells formed a confluent monolayer exhibiting typical cobblestone morphology without preferential orientation (Figure 3). 

Graft patency and diameter changes

**Figure 4:**
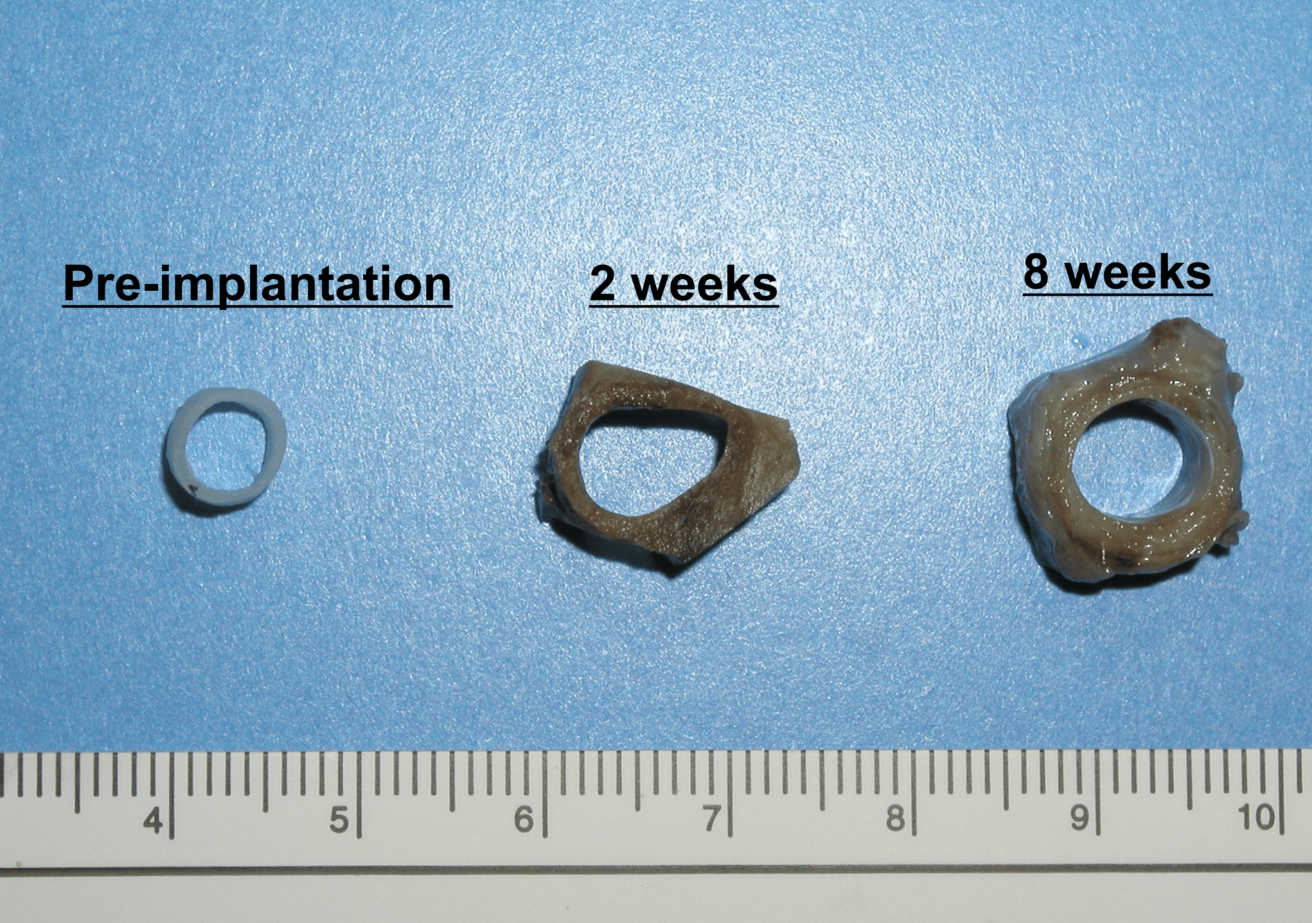
Gross appearance of the hybrid vascular graft before and after implantation. Representative macroscopic findings of the graft before implantation and after two and eight weeks of implantation; mild luminal dilatation was observed at two weeks compared with the preimplantation graft. No substantial additional dilatation was observed between two and eight weeks. At eight weeks, the surrounding tissue appeared denser and more organized than at two weeks.

**Figure 5:**
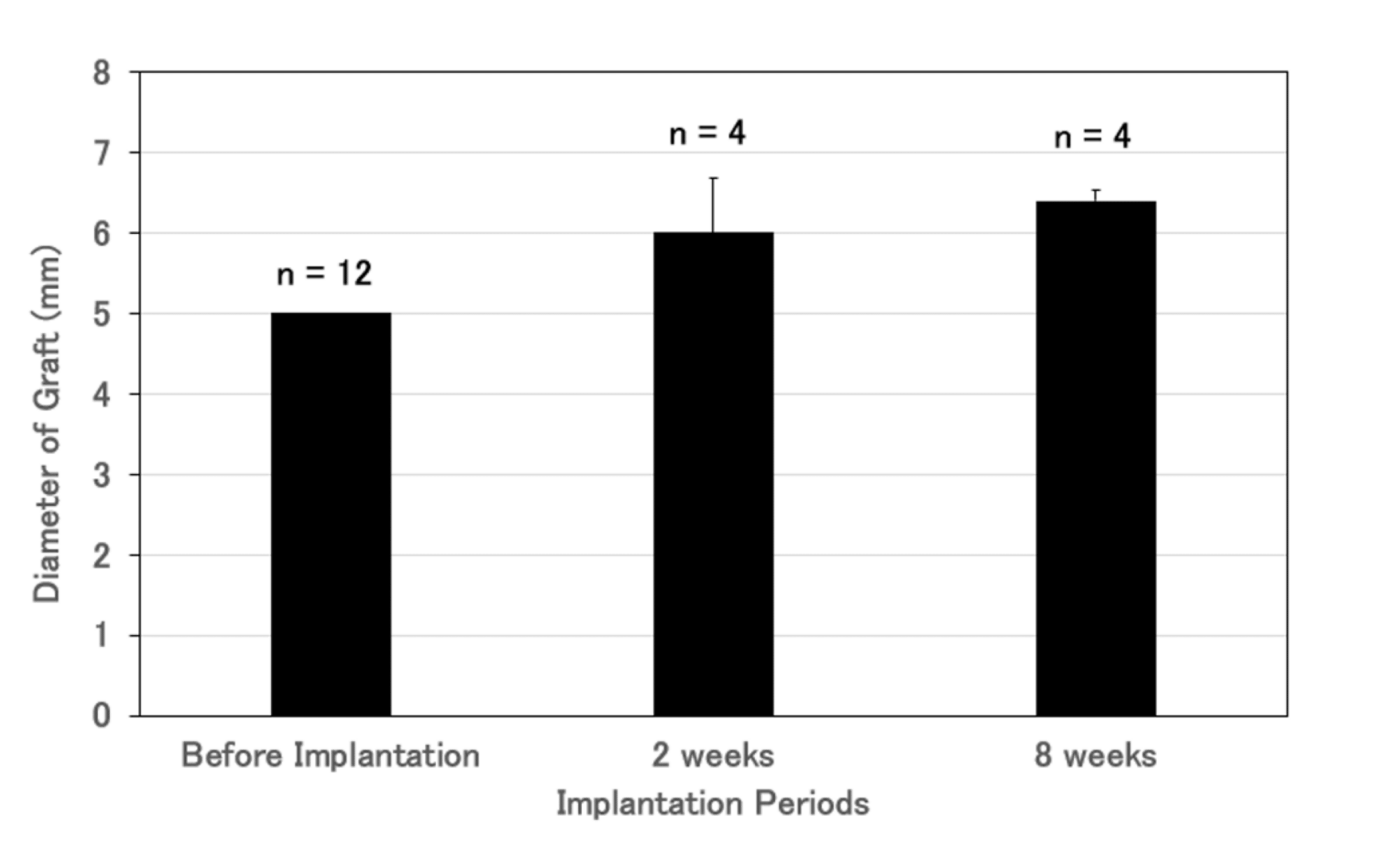
Changes in graft diameter after implantation. Quantitative analysis of graft diameter before implantation and after explantation; graft diameter increased significantly at two weeks after implantation and remained stable thereafter. Data are presented as mean ± standard deviation. Statistical analysis was performed using one-way ANOVA (F=28.37, P < .001), followed by two-tailed Student's t-tests for pairwise comparisons. Statistical significance was defined as P < .05.

Hybrid vascular grafts were implanted into the carotid arteries of the same animals from which autologous vascular cells had been harvested and were explanted after two weeks (n = 4) or eight weeks (n = 4). All hybrid grafts remained patent throughout the observation period. No gross evidence of thrombosis, aneurysmal degeneration, or graft rupture was observed at explantation (Figure 4). In contrast, all control grafts failed within one week after implantation. All control animals developed rapidly enlarging cervical hematomas and reached predefined humane endpoints. Emergency explantation demonstrated anastomotic dehiscence and/or rupture of the graft wall with massive hemorrhage. Quantitative image analysis demonstrated a significant increase in graft diameter from 5.0 mm before implantation to 6.0 ± 0.67 mm at two weeks. No additional significant enlargement was observed thereafter, and graft diameter remained stable at 6.4 ± 0.12 mm at eight weeks (Figure 5). 

Histological remodeling and scaffold degradation

**Figure 6:**
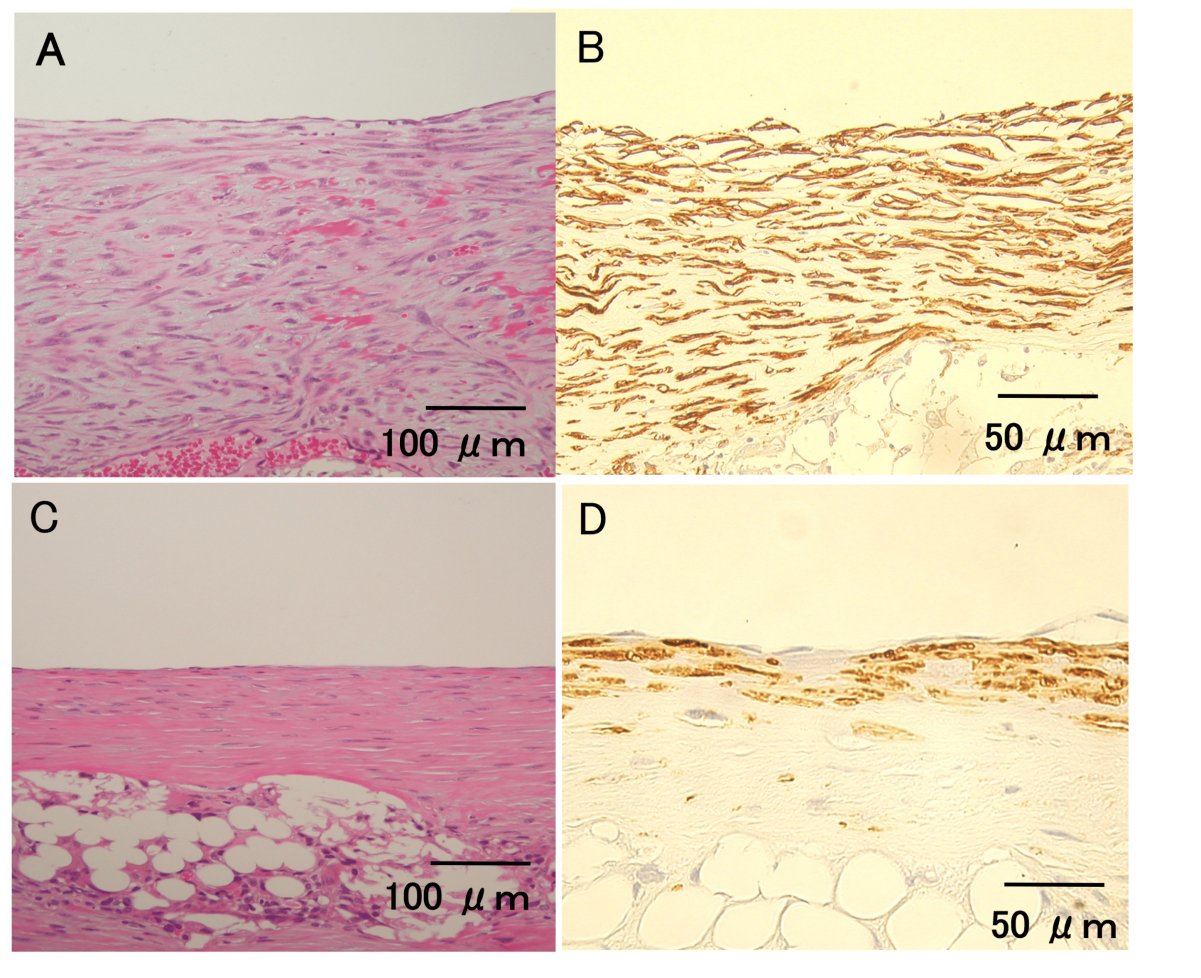
Histological remodeling of the graft wall and scaffold degradation after implantation. (A, B) Hematoxylin-eosin staining at two and eight weeks after implantation, respectively. (C, D) Immunohistochemical staining using hybridoma human fetus clone 35 (HHF-35), a monoclonal antibody recognizing muscle-specific α- and γ-actin isoforms. At two weeks, the graft wall was markedly thickened and contained numerous vascular wall cells embedded within abundant extracellular matrix. Most of the biodegradable polymer scaffold was no longer identifiable, whereas residual mesh fibers remained within the outer portion of the graft wall. Numerous HHF-35–positive smooth muscle cells were observed throughout the regenerated tissue. At eight weeks, the graft wall exhibited a more organized layered architecture with reduced wall thickness. The biodegradable polymer scaffold had completely disappeared and was replaced by regenerated vascular tissue. In contrast, mesh fibers remained identifiable within the outer layer of the graft wall. Smooth muscle cells accumulated beneath the endothelial layer, whereas fibroblasts were predominantly localized in the outer region, resulting in a structure resembling that of a native artery.

Two weeks after implantation, the regenerated tissue consisted of abundant extracellular matrix containing numerous SMCs and FCs. The graft wall was markedly thickened, and extensive cellular infiltration and neovascularization originating from the outer layer were observed. Most of the biodegradable polymer scaffold was no longer identifiable, whereas residual mesh fibers remained within the outer portion of the graft wall (Figures 6A, 6B).

By eight weeks, the regenerated tissue exhibited a highly organized layered architecture resembling that of native arteries. A confluent endothelial lining was present on the luminal surface, beneath which circumferentially aligned SMCs formed a multilayered medial structure. FCs were predominantly localized within the outer layer. At this stage, the polymer scaffold had completely disappeared and had been replaced by regenerated vascular tissue, whereas the reinforcing mesh fibers remained identifiable within the outer wall (Figures 6C, 6D). 

Changes in neoarterial wall thickness

**Figure 7:**
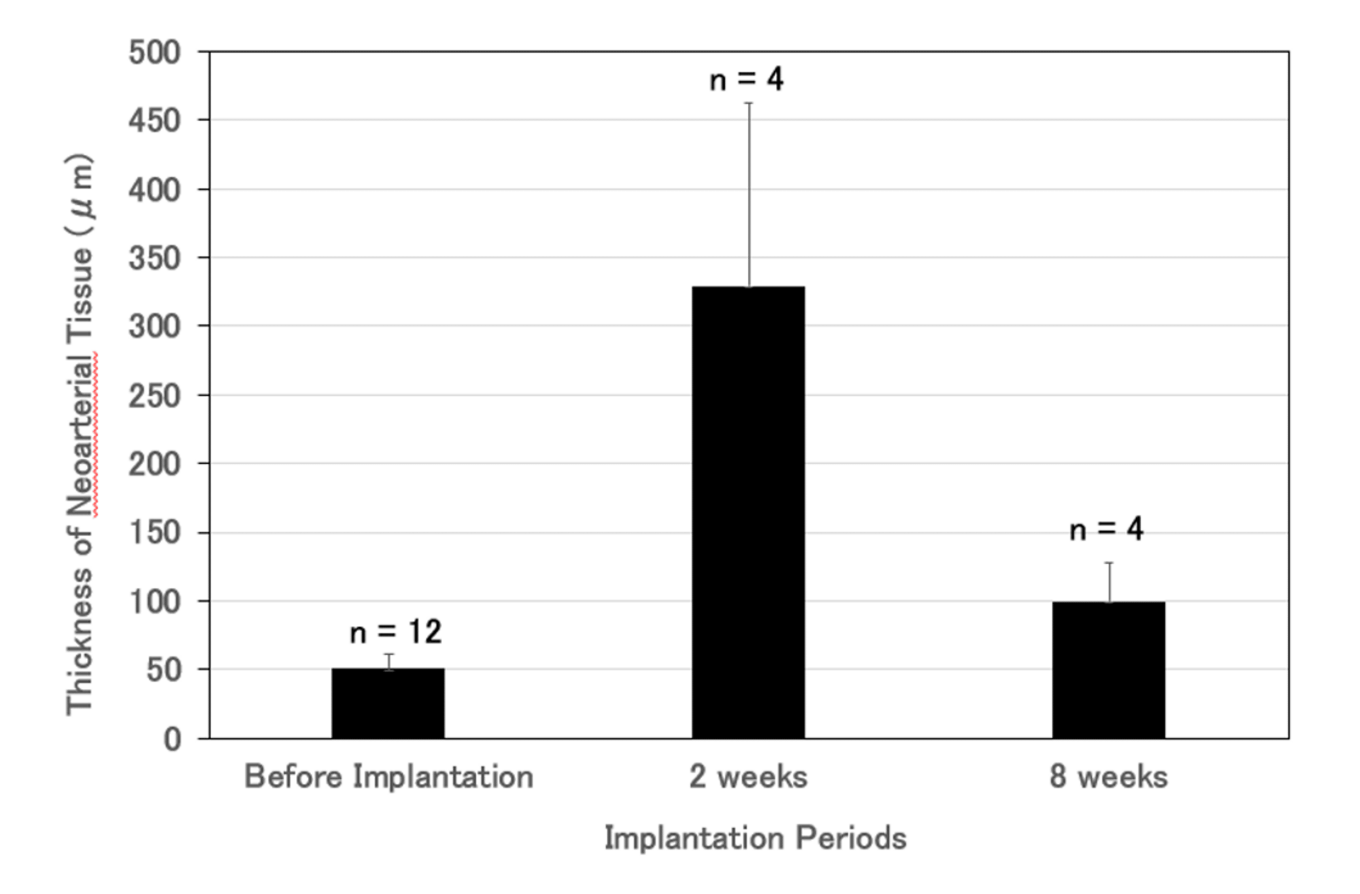
Temporal changes in neoarterial wall thickness. Morphometric analysis of neoarterial wall thickness before implantation and after explantation. Wall thickness increased significantly at two weeks after implantation and subsequently decreased significantly by eight weeks. Data are presented as mean ± standard deviation. Statistical analysis was performed using one-way ANOVA (F=24.47, P < .001), followed by two tailed Student's t-tests for pairwise comparisons. Statistical significance was defined as P < .05.

The thickness of the regenerated vascular wall changed significantly over time. Before implantation, the mean wall thickness was 50.8 ± 10.0 μm. At two weeks, wall thickness increased significantly to 329 ± 133 μm. By eight weeks, wall thickness decreased significantly to 99.9 ± 28.4 μm, although it remained greater than that observed before implantation (Figure 7). 

Endothelial coverage

**Figure 8:**
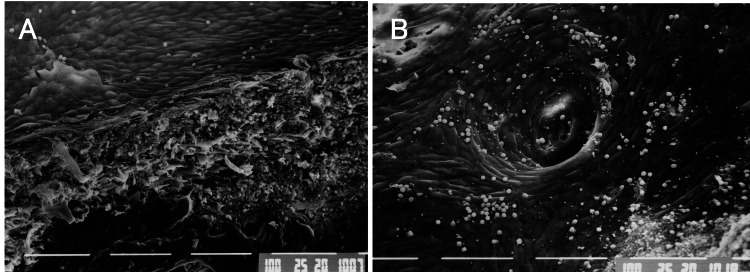
Scanning electron microscopic findings of the luminal surface after implantation. (A) At two weeks, the luminal surface was completely covered by endothelial cells aligned in the direction of blood flow. (B) At eight weeks, complete endothelial coverage was maintained, and openings of microvascular structures were observed on the luminal surface.

SEM demonstrated complete endothelial coverage of the luminal surface in all grafts examined at both implantation periods. At two weeks, endothelial cells formed a tightly interconnected monolayer aligned in the direction of blood flow (Figure 8A). At eight weeks, endothelial coverage remained complete, and openings of microvascular structures were observed on the luminal surface (Figure 8B). 

Ultrastructural evaluation of SMC phenotype

**Figure 9:**
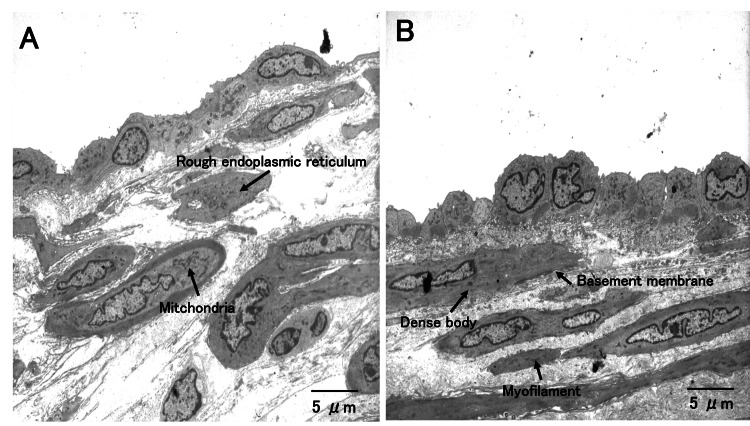
Ultrastructural changes in smooth muscle cell phenotype. Transmission electron microscopic findings of the graft wall after implantation. (A) At two weeks, most smooth muscle cells exhibited features characteristic of the synthetic phenotype, including abundant rough endoplasmic reticulum and mitochondria. (B) At eight weeks, smooth muscle cells demonstrated features of the contractile phenotype, including elongated morphology, prominent myofilaments, dense bodies, and well-developed basement membranes beneath the endothelial layer.

TEM revealed marked temporal changes in smooth muscle cell morphology. At two weeks, the majority of SMCs exhibited features characteristic of the synthetic phenotype, including abundant rough endoplasmic reticulum, free ribosomes, and mitochondria (Figure 9A).

At eight weeks, most SMCs demonstrated morphological characteristics of the contractile phenotype. These cells were elongated and contained abundant myofilaments arranged parallel to the long axis of the cell. Dense bodies and well-developed basement membranes were readily identified, whereas rough endoplasmic reticulum and free ribosomes were markedly reduced (Figure 9B). 

## Discussion

The present study demonstrated that hybrid tissue-engineered vascular grafts composed of autologous ECs, SMCs, and FCs remained patent in the arterial circulation and underwent progressive remodeling toward a native artery-like structure. The principal findings were: (1) complete endothelialization was maintained throughout the observation period; (2) the biodegradable polymer scaffold was almost completely degraded by two weeks and entirely absent by eight weeks; (3) the regenerated tissue progressively acquired a layered architecture resembling that of native arteries; and (4) SMCs underwent phenotypic transition from a synthetic to a contractile state during vascular wall maturation.

Despite decades of investigation, the development of durable small-diameter vascular grafts, typically defined as conduits with an internal diameter of ≤6 mm, remains an unresolved challenge [1]. Surface modifications such as heparin bonding and other antithrombotic coatings may improve short-term patency [11], but none can permanently reproduce the dynamic antithrombotic and antiplatelet functions of living ECs. Consequently, rapid endothelialization remains a fundamental prerequisite for long-term graft success.

However, endothelialization alone is unlikely to provide durable vascular regeneration. In native vessels, ECs depend on a specialized extracellular matrix and continuous interactions with SMCs and FCs. These supporting cells synthesize collagen, elastin, and other extracellular matrix components that provide both mechanical support and biological signaling. Therefore, successful regeneration requires reconstruction of the entire vascular wall rather than the luminal surface alone [4]. The catastrophic early failure of all control grafts further emphasizes this concept. Control grafts containing extracellular matrix alone developed massive hemorrhagic complications within one week after implantation, resulting from anastomotic dehiscence and/or rupture of the graft wall. In contrast, all hybrid grafts remained patent despite a rapid degradation of the polymer scaffold. These findings suggest that scaffold materials and extracellular matrix alone are insufficient to maintain structural integrity in the arterial circulation and that preconstruction of a cellular vascular wall is essential for successful arterial regeneration.

The present study focused on two specific time points representing the early and intermediate phases of vascular regeneration. In our previous study using polyester-based hybrid vascular grafts, a native artery-like wall structure had already developed by 12 weeks after implantation [10]. The eight-week time point was selected to determine whether biological tissue formation could compensate for scaffold degradation and establish a native artery-like wall before the complete disappearance of the polymer scaffold. The two-week time point was chosen to characterize the early remodeling process, particularly scaffold degradation and the ability of the graft to maintain structural integrity under arterial hemodynamic conditions. The present findings support this concept. Two weeks after implantation, the regenerated tissue was characterized by marked wall thickening, abundant extracellular matrix, and numerous synthetic SMCs. This early phase likely reflects active vascular wall construction. By eight weeks, the regenerated tissue had reorganized into a layered structure closely resembling native arteries, with ECs lining the lumen, SMCs forming a medial layer, and FCs localized predominantly in the outer wall.

A particularly important finding was the rapid disappearance of the biodegradable polymer scaffold. Histological examination demonstrated that most polymer components were no longer detectable by two weeks and had completely disappeared by eight weeks. In contrast, the absorbable mesh fibers remained present throughout the study period and likely contributed to the maintenance of mechanical stability during tissue remodeling.

These observations suggest that the polymer scaffold functioned primarily as a temporary regenerative template rather than a permanent structural component, consistent with the concept of scaffold-guided vascular regeneration proposed by Roh and colleagues [12].

By the time the scaffold had disappeared, the regenerated tissue had already developed a mature vascular architecture consisting of ECs, SMCs, FCs, and extracellular matrix. Moreover, smooth muscle cells had undergone phenotypic maturation toward a contractile state. Collectively, these findings indicate that biological tissue formation progressed rapidly enough to compensate for scaffold degradation even under arterial hemodynamic conditions.

The ultrastructural findings further support this interpretation. At two weeks, SMCs exhibited abundant rough endoplasmic reticulum, free ribosomes, and mitochondria, indicating active extracellular matrix synthesis. Such synthetic SMCs are essential during the early phase of vascular regeneration. However, prolonged persistence of this phenotype may result in excessive neointimal formation and graft failure. By eight weeks, most SMC demonstrated the characteristic features of the contractile phenotype, including elongated morphology, abundant myofilaments, dense bodies, and well-developed basement membranes. This transition is widely regarded as a hallmark of vascular wall maturation, and vascular stabilization suggests that the regenerated tissue had progressed from a proliferative state to a more stable and functional arterial wall [13,14].

The present findings extend previous observations reported by Shin’oka and colleagues and by Hibino and colleagues using biodegradable tissue-engineered vascular grafts [6,15]. Those studies demonstrated scaffold-guided tissue regeneration and subsequent remodeling, primarily in low-pressure venous or right-sided cardiovascular environments. In contrast, the present study demonstrates complete replacement of a biodegradable scaffold by organized arterial tissue under systemic arterial pressure. The persistence of structural integrity despite the disappearance of the scaffold suggests that vascular wall reconstruction had progressed sufficiently to support arterial hemodynamic loading.

One of the most attractive theoretical advantages of regenerative vascular grafts is the potential for somatic growth, particularly in pediatric cardiovascular surgery [16]. Although long-term patency and somatic growth are among the ultimate clinical goals of vascular tissue engineering, we believe that these outcomes depend fundamentally on the establishment of a living vascular wall. Therefore, the primary focus of the present study was to determine whether a biodegradable scaffold could be replaced by biologically organized arterial tissue capable of supporting long-term remodeling under arterial hemodynamic conditions. Recent studies have further demonstrated that tissue-engineered vascular grafts can transform into autologous neovessels capable of native vascular function and growth, highlighting the potential for patient-specific regenerative therapies in the future [17].

Although growth capacity was not directly evaluated in the present study, the formation of a mature artery-like wall following scaffold degradation suggests that the regenerated conduit may behave more like living vascular tissue than a conventional prosthetic graft. Because the reinforcing mesh fibers remained present at 8 weeks, longer-term studies are required to determine whether vascular integrity can be maintained after complete mesh resorption and whether adaptive growth occurs thereafter.

Several limitations should be acknowledged. First, the number of animals was relatively small, consisting of four animals in each hybrid graft group and four control animals. Second, the observation period was limited to eight weeks. This time point was selected because the primary objective of this study was to evaluate early scaffold degradation and vascular wall maturation under arterial hemodynamic conditions. In our previous study using polyester-based hybrid vascular grafts, a native artery-like wall had already developed by 12 weeks after implantation [10]. Therefore, we hypothesized that vascular maturation might occur earlier with the present biodegradable scaffold and selected eight weeks as the endpoint for evaluating scaffold replacement and arterial wall regeneration.

The relatively small sample size and limited duration of follow-up were also influenced in part by increasing restrictions on the use of large-animal experimental facilities arising from institutional and societal changes regarding animal experimentation, which precluded expansion of the study population and longer follow-up.

Nevertheless, the selected time points captured the critical stages of vascular regeneration, including scaffold degradation, vascular wall remodeling, and SMC phenotypic maturation. Future studies with larger cohorts and longer observation periods are required to determine whether vascular integrity, long-term patency, and growth potential are maintained after complete degradation of the remaining mesh fibers.

In addition, the present study did not include comparisons with conventional synthetic prostheses or other tissue-engineered vascular grafts. The primary objective of this study was to investigate the biological process of scaffold replacement and vascular wall maturation in a hybrid graft under arterial hemodynamic conditions rather than to compare different graft materials or strategies. Future studies directly comparing hybrid grafts with currently available prosthetic grafts and alternative tissue-engineered approaches are warranted.

## Conclusions

In conclusion, hybrid tissue-engineered vascular grafts promoted rapid endothelialization, progressive vascular wall maturation, and replacement of a biodegradable scaffold by organized arterial tissue. The formation of a native artery-like wall despite scaffold degradation suggests that the graft functioned as a temporary regenerative template rather than a permanent prosthesis. These findings support the feasibility of generating a living arterial conduit and suggest that reconstruction of a native artery-like wall may provide the biological foundation for both long-term patency and future growth.
